# Association between SARS-CoV-2 infection and disease severity among prostate cancer patients on androgen deprivation therapy: a systematic review and meta-analysis

**DOI:** 10.1007/s00345-021-03810-6

**Published:** 2021-09-03

**Authors:** Reza Sari Motlagh, Mohammad Abufaraj, Pierre I. Karakiewicz, Pawel Rajwa, Keiichiro Mori, Dong-Ho Mun, Shahrokh F. Shariat

**Affiliations:** 1 Department of Urology, Comprehensive Cancer Center, Vienna General Hospital, Medical University of Vienna, Währinger Gürtel 18-20, 1090 Vienna, Austria; 2 Men’s Health and Reproductive Health Research Center, Shahid Beheshti University of Medical Sciences, Tehran, Iran; 3 Department of Special Surgery, Jordan University Hospital, The University of Jordan, Amman, Jordan; 4 The National Center for Diabetes, Endocrinology and Genetics, The University of Jordan, Amman, Jordan; 5 Cancer Prognostics and Health Outcomes Unit, University of Montreal Health Center, Montreal, Canada; 6 Department of Urology, Medical University of Silesia, Zabrze, Poland; 7 Department of Urology, The Jikei University School of Medicine, Tokyo, Japan; 8 Institute for Urology and Reproductive Health, Sechenov University, Moscow, Russia; 9 Department of Urology, Weill Cornell Medical College, New York, NY USA; 10 Department of Urology, University of Texas Southwestern, Dallas, TX USA; 11 Second Faculty of Medicine, Department of Urology, Charles University, Prague, Czech Republic; 12 Karl Landsteiner Institute of Urology and Andrology, Vienna, Austria

**Keywords:** Androgen deprivation therapy, ADT, COVID-19, Disease severity, SARS-CoV-2, Infection risk, Prostate cancer

## Abstract

**Purpose:**

Androgen-regulated enzymes such as the angiotensin-converting enzyme 2 (ACE2) and the transmembrane serine protease 2 (TMPRSS2) are involved in the SARS-CoV-2 infection process. The expression of TMPRSS2 and its fusion gene, which are increased in the epithelium of the human prostate gland during prostate carcinogenesis, are regulated by androgens. Our goal was to assess the risk of the SARS-CoV-2 infection and the severity of the disease in PCa patients treated with androgen deprivation therapy (ADT).

**Methods:**

We conducted a systematic review and meta-analysis according to PRISMA guidelines. We queried PubMed and Web of Science databases on 1 July 2021. We used random- and/or fixed-effects meta-analytic models in the presence or absence of heterogeneity according to Cochrane’s *Q* test and *I*^2^ statistic, respectively.

**Results:**

Six retrospective studies (*n* = 50,220 patients) were selected after considering inclusion and exclusion criteria for qualitative evidence synthesis. Four retrospective studies were included to assess the SARS-CoV-2 infection risk in PCa patients under ADT vs. no ADT and the summarized risk ratio (RR) was 0.8 (95% confidence intervals (CI) 0.44–1.47). Five retrospective studies were included to assess the severity of coronavirus disease 2019 (COVID-19) in PCa patients under ADT versus no ADT and the summarized RR was 1.23 (95% CI 0.9–1.68).

**Conclusion:**

We found a non-significant association between the risk of SARS-CoV-2 infection and COVID-19 severity in PCa patients treated with ADT. However, our results suggest that during the COVID-19 pandemic PCa patients can safely undergo ADT as a cancer therapy without worsening COVID-19 risk and trajectory.

## Introduction

The incidence of SARS-CoV-2 infection is equal in both sexes; however, disease severity and progression rates are approximately three times higher in the male gender [1–3]. This sex-specific discrepancy can potentially be explained by the mechanism of SARS-CoV-2 entry into human host cells. Both enzymes, the transmembrane angiotensin-converting enzyme 2 (ACE2) and the transmembrane protease, serine 2 (TMPRSS2) regulate SARS-CoV-2 invasion through cell membrane [4, 5]. These enzymes are androgen-dependent and can be strongly upregulated by elevated levels of androgens [4, 6]. Moreover, the expression of TMPRSS2 is found in the lung, gastrointestinal system, and heart [4, 6]. The expression of TMPRSS2 and its fusion gene, which are increased in the epithelium of the human prostate gland [4] during prostate carcinogenesis, are regulated by androgens. Indeed, TMPRSS2–ERG (erythroblast-specific-related gene) gene fusion is one of the best-known aberrations in PCa [4] with overexpression detected in about 40–50% of PCa patients [7].

Androgen deprivation therapy (ADT) and the second-generation androgen receptor (AR) targeting therapy were developed to suppress the androgen-activated intracellular cascade that leads to tumor progression and aggressive tumor growth [6]. Several trials are currently evaluating androgen suppression in patients with SARS-CoV-2 infection with a focus on coronavirus disease 2019 (COVID-19) disease severity [8–12]. Since such associations can only be uncovered by very large datasets/cohorts, we aimed to aggregate data through systematic review and meta-analysis to assess the risk of SARS-CoV-2 infection and the severity of disease in PCa patients treated with ADT. Understanding either patients on ADT are at higher risk of SARS-CoV-2 infection and severe COVID-19 or there is a protective effect of ADT.

## Methods

### Literature search

A protocol for this study was registered a priori on the International Prospective Register of Systematic Reviews (CRD42021249405). We followed the preferred reporting items for systematic reviews and meta-analysis (PRISMA) guidelines. PubMed and Web of Science were used to search for specific queries on 1 July 2021. The search query lines and strategies were *“(((ADT) OR (“androgen deprivation therapy”[All Fields])) AND (“SARS-CoV-2”[All Fields]))* in PubMed database and *“ALL* = *((“androgen deprivation therapy” OR “ADT”) AND (“SARS-CoV-2” OR “COVID-19”))*” in Web of Science database.

### Inclusion/exclusion criteria

We only retrieved original studies published in English and excluded all other types of reports. Our main objective was to test the hypothesis stating that PCa patients who received ADT might have a lower risk of SARS-CoV-2 infection and experience a less severe form of the disease. The PICO framework items were: P (population) PCa patients and SARS-CoV-2 positive PCa patients; I (intervention group) ADT; C (control group) non-ADT; O, (outcomes) the SARS-CoV-2 infection and severe form of the disease. The severe disease was defined as ICU admission, intubation, and/or COVID-19 death. All current articles that assessed the risk of SARS-CoV-2 infection among PCa patients were eligible for this systematic review. We did not restrict our inclusion criteria to specific ADT, therefore studies analyzing GnRH agonist and antagonist, as well as oral antiandrogens, were eligible. Inclusion criteria for the quantitative meta-analysis involved all original research articles including cohort, case–control, and randomized control studies that assessed the overall risk of SARS-CoV-2 infection and severe disease as outcomes (ADT) with a control group that consisted of no ADT. Exclusion criteria involved studies without a control group (ADT).

### Data extraction

Two reviewers screened the article titles and abstract screening and any disagreements about eligible and ineligible articles were resolved according to Delphi consensus criteria between co-authors. We used a data extraction sheet developed based on the Cochrane Consumers and the Communication Review Group’s data extraction template (http://cccrg.cochrane.org/author-resources). We extracted the following data: first-author, type of article, year of publication, dates of the data collection or enrollment, study design, sample size, number of individuals in each study group, outcomes, how the outcomes were measured, follow-up duration, type of effect statistic and corresponding *p *value. In the case of lacking data or doubts, we contacted articles’ corresponding author(s) for additional details to overcome data limitations.

### Statistical analysis

Forest plots were used to calculate and graphically depict risk ratio (RR) and summarized them to describe the RR of the SARS-CoV-2 infection and severe disease rates in the treatment and control groups. Primary and secondary meta-analyses were conducted among all studies that reported the SARS-CoV-2 infection and/or severe disease rates as an outcome. The heterogeneity across studies was evaluated using Cochrane’s *Q* test and *I*^2^ statistics [13]. Significant heterogeneity was indicated by a *p* ≤ 0.05 in Cochrane’s *Q* tests and a ratio ≥ 50% in *I*^2^ statistics. We used fixed-effects models to calculate non-heterogeneous results. Random effect models were used in cases of heterogeneity. *p* values lower than 0.05 were considered statistically significant. All analyses were carried out using Cochrane Collaboration Review Manager software (RevMan v.5.4; Cochrane Collaboration, Oxford, UK).

### Risk of bias

Modified Newcastle–Ottawa Scale criteria were used to assess the quality of the included retrospective studies [14]. Above 6 points studies were considered as fair and good quality. Moreover, we used the Agency for Healthcare Research and Quality (AHRQ)[15]. The treatment and control groups of four studies were adjusted according to the potential confounding factors such as age, ischemic heart disease (IHD), hypertension, diabetes mellitus (DM), chronic obstructive pulmonary disease (COPD), and smoking status [16–19]. Montopoli et al. analyzed only age-adjusted data and their methodology was different from the other studies [20]. While all studies but Montopoli et al. divided the number of positive SARS-CoV-2 PCa patients (on or off ADT) into all SARS-CoV-2 tested PCa patients (on or off ADT), Montopoli et al. use prevalent cancer patients’ data of the region registry[20]. The risk of bias and quality assessment of all studies included in the meta-analysis are summarized in Table 1.

**Table 1:** The Newcastle–Ottawa Scale for all studies in quantitative synthesis

Study	Selection	Compatibility	Outcome	Total	AHRQ standards
Klein et al. [19]	****	**	**	8	Good
Montopoli et al. [20]	**	**	**	6	Fair
Koskinen et al. [16]	****	**	**	8	Good
Kwon et al. [17]	****	**	**	8	Good
Patel et al. [18]	****	**	*	7	Good

*AHRQ *Agency for Healthcare Research and Quality

Each asterisk (*) represents an individual criterion within the subsection that was fulfilled

## Results

### Selection process

**Fig. 1:**
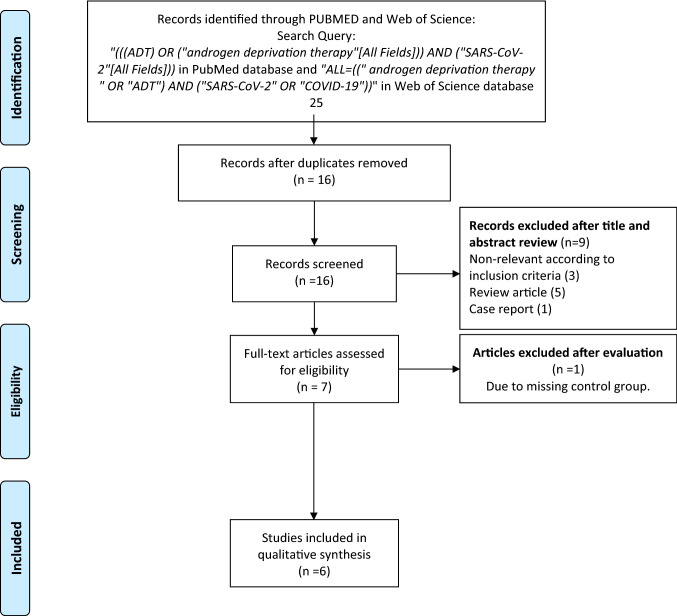
The selection process of the articles to assess the infection risk and severity of disease among prostate cancer patients who received androgen deprivation therapy compared to those who did not receive

After initial screening and excluding the duplicates, 16 articles were selected for further assessment. The search string is shown in Fig. 1. After applying the inclusion and exclusion criteria six studies (Patel et al. updated their study in 2021 [18, 21]) were included for systematic review; Caffo et al. was excluded from the meta-analysis due to the lack of a control group [22].

### Characteristics of included studies

Table 2 illustrates the characteristics of the five included studies. Four and five studies reported the risk of SARS-CoV-2 infection and severe COVID-19, respectively [16–21]. The reported outcomes including the odds ratio or hazard ratio of the SARS-CoV-2 infection and severe disease risks from the included studies are mentioned in Table 3.

**Table 2:** Characteristics of included studies in the qualitative and quantitative synthesis to assess the risk of infection and the severity of disease among PCa

Study/year	Design	Total patients	ADT group	Non-ADT group	Outcomes
Klein et al. 2021, United State [19]	Prospective cohort	1779	304	1475	Infection risk Severity of disease
Montopoli et al. 2020, Italy [20]	Retrospective cohort	42,434	5273	37,161	Infection risk Severity of disease
Koskinen et al. 2020, Finland [16]	Retrospective cohort	352	134	218	Infection risk Severity of disease
Kwon et al. 2020, United State [17]	Retrospective cohort	5211	799	4412	Infection risk Severity of disease
Patel et al. 2020 and 2021 United State^a^ [18]	Retrospective cohort	58 and 465	22 and 148	36 and 317	Severity of disease

Patients who received ADT compared to who did not receive ADT

*ADT* androgen deprivation therapy, *PCa* prostate cancer

^a^This study is updated in 2021

**Table 3:** The reported outcomes of the included studies regarding the infection risk and severity of disease among prostate cancer patients who received ADT compared to who did not receive ADT

Study/year/country	Infection risk for ADT	Disease severity
Klein et al. 2021, United State [19]	OR: 0.9; 95% CI 0.54–1.61, *p* = 0.8	Sample size limitations
Montopoli et al. 2020, Italy [20]	OR: 4.05; 95% CI; 1.55–10.59, *p*** = 0.0043**	OR: 4.40; CI 0.76–25.50, *p* = 0.0982
Koskinen et al. 2020, Finland [16]	OR: 0.88; 95% CI 0.32–2.44, *p* = 0.81	OR: 0.53; 95% CI 0.04–6.66, *p* = 0.63
Kwon et al. 2020, United State [17]	OR: 1.30; 95%CI 0.78–2.19, *p* = 0.31	OR: 0.56, 95% CI 0.07–4.88, *p* = 0.60
Patel et al. 2020, United State [18]	N/A	Death, OR: 0.37; 95% CI 0.08–1.80, *p* = 0.220
		Intubation, OR: 0.31; 95%CI 0.05–1.81, *p*=0.192
	N/A	Overall survival, HR 1.28; 95% CI 0.79–2.08, *p* = 0.32
		Intubation, HR 1.07; 95% 0.51–2.23, *p* = 0.87

*ADT* androgen deprivation therapy, *OR* odds ratio, *HR* hazard ratio

### Meta-analysis

#### Role of SARS-CoV-2 infection risk according to ADT use

**Fig. 2:**
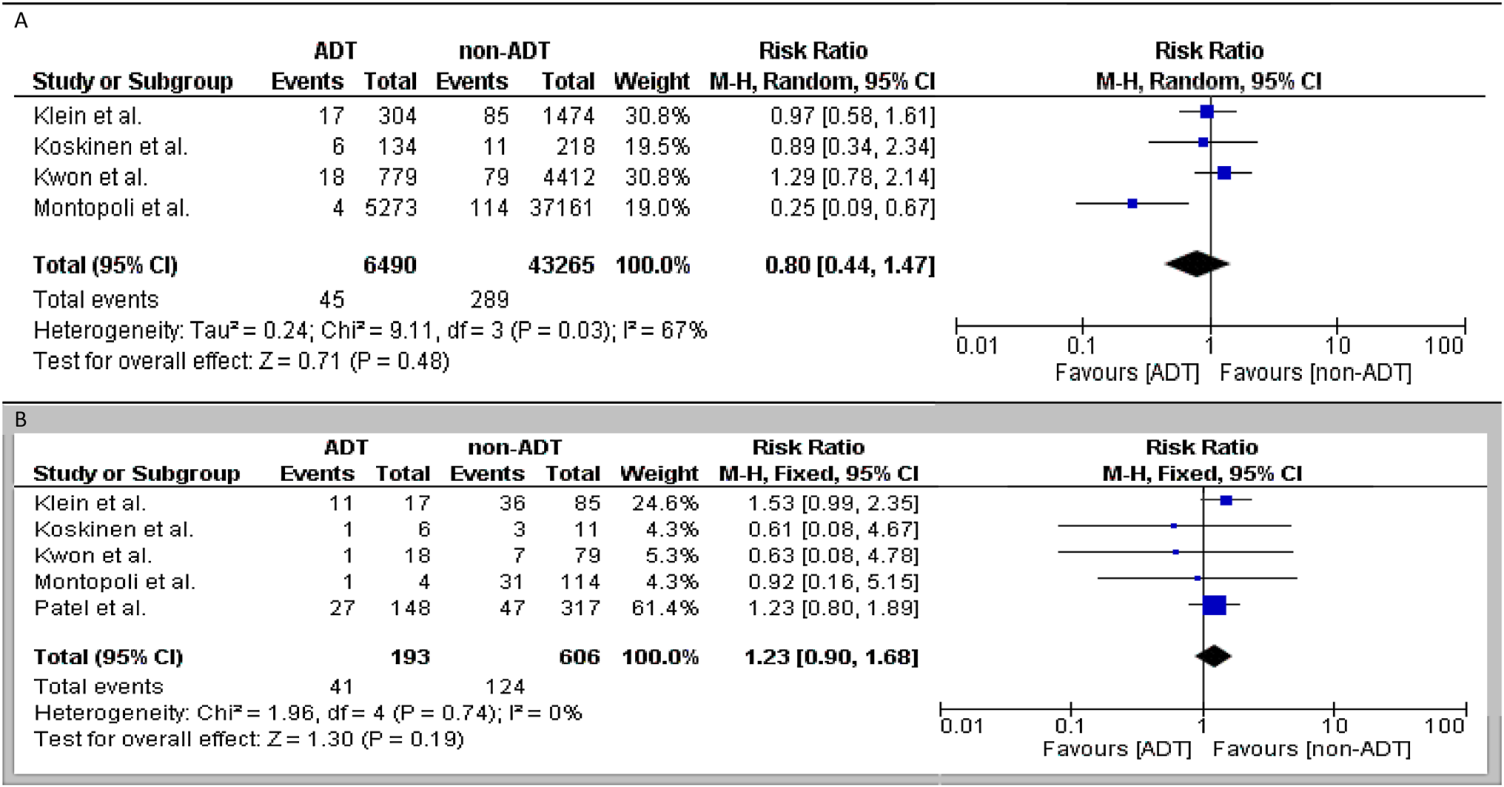
Forest plot, Relative risk (RR) of the infection risk (**A**) and severity of disease (**B**) among prostate cancer patients who received ADT compared to those who did not receive ADT. ADT: androgen deprivation therapy

We performed a meta-analysis, of the studies that compared the SARS-CoV-2 infection rate between ADT and no ADT PCa patients. The summarized RR of the four retrospective studies that assessed the SARS-CoV-2 infection risk (primary outcome) was 0.8 (95% confidence intervals (CI) 0.44–1.47; *p* = 0.48). The heterogeneity was high (*I*^2^ = 67%, *p* = 0.03), so a random effect model was used. After excluding the study of Montopoli et al. that reported different results compared to the other studies, the heterogeneity decreased (*I*^2^ = 0%, *p* = 0.67); the summarized RR of the three remaining studies remained statistically non-significant (RR 1.08, 95% CI 0.77–1.51; *p* = 0.64). The Forest plots of the meta-analysis are shown in Fig. 2A.

#### SARS-CoV-2 disease severity according to ADT use

We performed a meta-analysis of the studies that compared the disease severity between ADT and no ADT PCa patients. The summarized RR of five retrospective studies that assessed disease severity (secondary outcome) was 1.23 (95% CI 0.9–1.68; *p* = 0.19). The five studies included in the meta-analysis had a low heterogeneity (*I*^2^ = 0%, *p* = 0.74), so a fixed effect model was used. The Forest plots of the meta-analysis are shown in Fig. 2B.

## Discussion

The present systematic review and meta-analysis of the current cohort studies did not find a significant association between ADT use and SARS-CoV-2 infection or COVID-19 disease severity in PCa patients. The analyzed RRs suggested an association between ADT use and the decreased risk of infection and increased severe disease; however, statistical significance was not reached. Although our results could not support the hypothesized protective effect of ADT against COVID-19 disease, there was also no increased risk of SARS-CoV-2 infection and disease severity among PCa patients under ADT. Therefore, the treatment of PCa patients with ADT during the COVID-19 pandemic might be safely conducted as a cancer therapy.

Our first meta-analysis result revealed high heterogeneity; thus, we tried to find the probable factors by assessment of the design of included studies. The first reports, which analyzed the SARS-CoV-2 infection risk among PCa patients under ADT suggested that androgen suppression could be protective against the SARS-CoV-2 [20]. Consequently, urological centers in the US and Finland conducted cohort studies to assess this potentially protective effect [16–19]. However, none of these could confirm a benefit to the use of ADT in reducing the risk of infection and severity of the disease. Four later cohort studies analyzed PCa patients in both ADT and no ADT groups in terms of the potential confounding factors such as IHD, COPD, hypertension, and DM; the study of Montopoli et al. did not [20]. While, Obesity, hypertension, DM, and heart failure have been identified as risk factors of poor outcomes in both SARS-CoV-2 patients [1]. We found a low heterogeneity in the subgroup meta-analysis after excluding the Montopoli study, while the association between SARS-CoV-2 infection and ADT remained insignificant. ADT, the mainstay treatment in advanced and metastatic PCa patients, is associated with several adverse events (AEs) such as cardiac diseases and metabolic syndrome, osteoporosis, fractures, and cognitive disorders. This, indeed, can affect PCa patients' performance status and overall survival [23–25]. O’Callaghan et al. calculated that to prevent one COVID-19 case, 434 men need to be treated with ADT [26]. Thus, ADT could not provide a feasible treatment option in comparison with potential side effects.

A high rate of hospitalization was reported among metastatic castration resistance prostate cancer (mCRPC) patients who suffered from SARS-CoV-2 infection, moreover, the mortality was significantly associated with the number of previous PCa treatment lines such as different ADTs and chemotherapy [22]. Thereby, the duration of ADT and stage of disease (e.g., advanced, metastatic, CRPC, metastatic CRPC) could be the unreported confounding factor in the cohort studies that assessed the risk of SARS-CoV-2 infection and the severity of COVID-19. It has been shown that after long-term ADT and in CRPC tumors, the activity of AR remains elevated, despite reduced circulating androgen levels. Likewise, TMPRSS2 and TMPRSS2-ERG fusion genes are highly prevalent in PCa patients, including CRPC [27, 28]. Nevertheless, the expression of TMPRSS2 and its fusion gene in the involved organs (e.g., lung, kidney, and heart) during the SARS-CoV-2 infection process have not been investigated among PCa patients treated with ADT.

The protective effect of androgen suppression has been hypothesized and case–control studies and RCTs made an effort to assess its potential effect. Up to date, the results of the RCTs are scarce to fully explore the androgen suppression effect on the treatment of the SARS-CoV-2 infection [8–10, 12]. Additionally, the reported results of studies that assessed the protective and/or therapeutic effect of 5-alpha reductase inhibitors (5ARIs) suffer from a small sample size, the inconsistency of results, and a selection bias [29–31]. While one RCT and one case–control study found that 5ARIs reduced viral shedding/ inflammatory markers in mild to moderate COVID-19 patients and the relative risk for severe disease [29, 30]. Another population-based case–control study with a larger sample size could not show such a protective effect against COVID-19 severity[31].

The main limitation of the present systematic review and meta-analysis was the few cohort studies that assessed the risk of SARS-CoV-2 infection and COVID-19 severity among PCa patients treated with ADT. However, owing to the challenges in study design with an infectious disease it is unlikely that a good design prospective clinical trial can be performed. Moreover, up to now no study queried and mentioned this important question, which level of androgen suppression and duration are needed to show protective and/or therapeutic effectiveness.

## Conclusions

We found a non‐significant association between the SARS-CoV-2 infection and disease severity with ADT use among PCa patients. Although our results could not support the protective effect of ADT against SARS-CoV-2 infection and disease severity, we found that ADT does not worsen COVID-19 risk and trajectory. Indeed, ADT as a cancer treatment might be safely administered to patients during the COVID-19 pandemic. A larger sample size with adjustment of the effects of all potential confounding factors such as duration of ADT and different stages of disease (i.e. CRPC and mCRPC) is necessary to further evaluate the impact of ADT on the risk and severity of SARS-CoV-2 infection. Moreover, the expression of ACE2 and TMPRSS2 in different organs and various stages of PCa in patients treated with ADT may help uncover the source of the biological rationale.
